# QT Prolongation Following Premature Ventricular Contractions Leading to Ventricular Storms: A Case Report

**DOI:** 10.7759/cureus.92554

**Published:** 2025-09-17

**Authors:** Khatuna Jalabadze, Bachuki Tsiklauri

**Affiliations:** 1 Cardiology, Geo Hospitals, Tbilisi, GEO; 2 Internal Medicine, Tbilisi Heart Centre, Tbilisi, GEO

**Keywords:** arrhythmia risk stratification, congenital long qt syndrome, implantable-cardioverter defibrillator, life threatening arrhythmia, post-pvc qtc prolongation, premature ventricular contractions, qt interval prolongation, torsades de pointes (tdp), variant of uncertain significance, ventricular tachycardia (vt) storm

## Abstract

QT interval behavior following premature ventricular contractions (PVCs) has not been widely studied, but it may serve as a valuable diagnostic and prognostic marker for malignant arrhythmias, particularly when standard QT assessment fails to provide clear answers. This report follows up on a previous case involving a 53-year-old woman with recurrent syncope and QTc prolongation following PVCs. She was initially diagnosed with long QT syndrome (LQTS) in 2007. Despite interventions, including implantation of an implantable cardioverter-defibrillator (ICD), high-dose beta-blockers, and cardiac sympathetic denervation, she continued to experience torsades de pointes, ventricular tachycardia storms, and multiple hospitalizations. Genetic testing revealed variants of uncertain significance (VUS), none of which, to our knowledge, have been functionally studied in vitro or reported in the existing literature. This case highlights the potential importance of post-PVC QT prolongation as a marker of arrhythmic risk and underscores the challenges in diagnosing and managing LQTS. Greater awareness of this phenomenon could enhance risk assessment in patients with unexplained syncope and borderline QT intervals. Further research is needed to determine its prognostic value and clinical relevance.

## Introduction

A prolonged QT interval on an ECG is a crucial indicator of cardiac repolarization abnormalities and a potential precursor to fatal arrhythmias [1]. Population studies indicate that normal QTc values range from 350 to 450 milliseconds (ms) for males and 360 to 460 ms for females. However, interpretation can be challenging due to the overlap in QT values between healthy individuals and those with electrical disorders such as long QT syndrome (LQTS) [2].

Premature ventricular contractions (PVCs) have also been identified as predictors of sudden death and malignant arrhythmias [3]. QT prolongation following PVCs is a recognized ECG phenomenon but remains poorly studied. Especially, there is no information regarding its relation to life-threatening ventricular tachycardia (VT) in LQTS. Some individuals exhibit a normal baseline QT interval that prolongs only after PVCs occur. Despite its limited discussion in the scientific literature, post-PVC QT interval behavior may play an important role in risk stratification, particularly in diagnostically ambiguous cases. This case report serves as a clinical example of that hypothesis and provides an insight into cardiac electrophysiology. It follows up on a previously published case [4] where the patient presented with recurrent arrhythmogenic syncope and demonstrated post-PVC QT interval prolongation, among other ECG findings. Since then, significant developments have occurred in the patient’s clinical course.

## Case presentation

**Figure 1:**
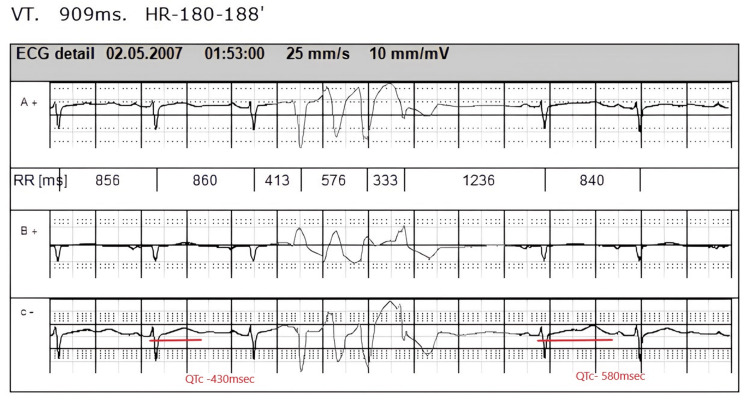
PVCs followed by QT interval prolongation in the subsequent sinus beat PVC: premature ventricular contraction

The patient, a 53-year-old woman, received a provisional diagnosis of long QT syndrome in 2007. She initially presented with recurrent episodes of syncope, with neurologic causes ruled out. Due to a positive family history of sudden death, including that of her paternal aunt and her sister, who died in her sleep, an extensive cardiologic evaluation was pursued. Notably, a 24-hour ECG revealed QT interval prolongation occurring specifically after premature ventricular contractions. According to Bazett's formula, the baseline corrected QT (QTc) interval was 430 ms; however, the QTc interval following the first sinus beat after a PVC ranged from 520 to 600 milliseconds (Figure 1). Additionally, a brief episode of non-sustained VT was observed. No QT abnormalities were noted during treadmill testing, and no structural changes were detected via echocardiography. Repeated measurements of electrolytes and thyroid panel values were within normal limits.

Due to ECG abnormalities, unexplained syncopal episodes, and a strong family history of sudden cardiac death (SCD), the patient was classified as high-risk for SCD. An implantable cardioverter-defibrillator (ICD) was implanted, and high-dose beta-blockers (metoprolol succinate 150mg per day initially with dose titration according to heart rate) were prescribed. Confirmatory genetic testing (on KCNQ1, KCNH2, SCN5A) was performed on blood samples from the patient and her family to detect common LQTS mutations, but no pathogenic variants were identified.

**Figure 2:**
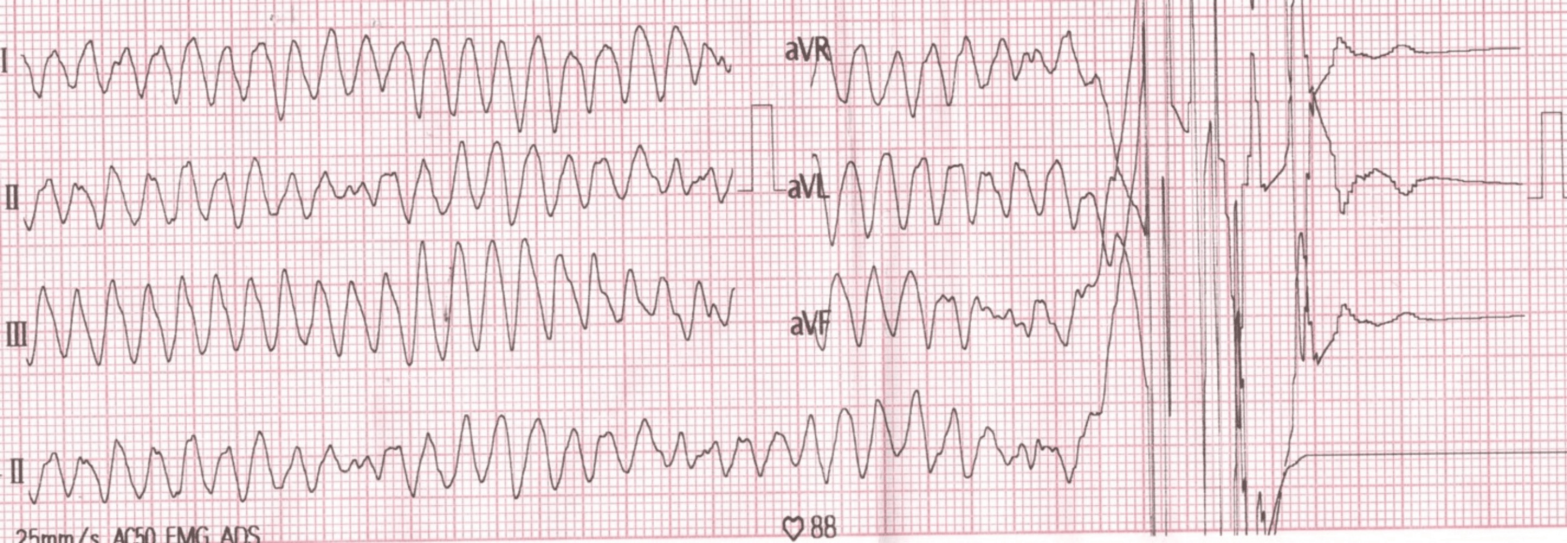
ECG: Episode of TdP TdP: torsades de pointes

The patient underwent regular follow-up over several years, during which her condition remained stable, with no major rhythm abnormalities, until May 2020. At that time, she was hospitalized for episodes of VT storm, including polymorphic ventricular tachycardia and torsades de pointes (TdP) (Figure 2). The arrhythmia was controlled through pacing, deep sedation, strict electrolyte management, and magnesium infusion. Notably, the potassium level was never below 3.9-4.2 mmol/L at admission. Coronary angiography revealed normal coronary arteries, and echocardiography demonstrated normal cardiac chambers, an ejection fraction (EF) of 56%, and no valvular disease.

The following year, despite switching from metoprolol to high-dose propranolol (as nadolol was unavailable in the country), the patient was again hospitalized due to a sustained VT storm. Cardiac sympathetic denervation was performed due to medication-refractory recurrent ventricular tachycardia and fibrillation. She continued propranolol at a dose of 80 mg every six hours.

Eight months after undergoing bilateral sympathetic denervation, the patient developed severe, recurrent episodes of ventricular tachycardia, resulting in more than 20 ICD-delivered shocks along with numerous short VT runs within a three-hour period. Over the course of three weeks, under deep sedation and close hemodynamic monitoring, the medical team successfully suppressed the arrhythmias. Subsequently, the ICD was replaced due to end-of-life (EoL) battery status following multiple shocks. A single-chamber generator was upgraded to a dual-chamber system to avoid symptomatic bradycardia due to high doses of propranolol. The pacemaker was programmed to avoid unnecessary ventricular pacing, with AAI to DDD mode activation and the atrial lower rate set to 85 beats per minute, to suppress VT, and for the patient's comfort. Additionally, she remained on maximal beta-blocker therapy (propranolol 240 mg daily).

Although initial genetic testing in 2008 yielded negative results, suspicion of LQTS persisted due to multiple subsequent episodes of life-threatening arrhythmias. As a result, more comprehensive genetic testing was performed (sequencing approach; Ion Torrent™ Personal Genome Machine) on a custom panel of genes that are most frequently associated with inherited arrhythmogenic diseases and cardiomyopathies. The panel provides a coverage of over 99% (IRCCS Pavia, Italy). The analysis identified several variants of uncertain significance (VUS). To our knowledge, these variants have not been functionally studied in vitro and have not been previously reported in the scientific literature (Table 1).

**Table 1:** Results of genetic screening for common LQTS mutations LQTS: long QT syndrome The displayed genetic variations were of uncertain pathogenic significance, and associated cases were not published in the scientific literature.

Gene	Variant			Pathogenicity	Reference
ASPH	c.1925A>G	p.Lys642Arg		VUS	Variant not published
LAMA4	c.4826A>G		p.Asn1609Ser	VUS	Variant not published
	c.2054C>T		p.Ser685Phe		

As of the most recent follow-up, the patient remains symptom free, with no rhythm disturbances or QT abnormalities observed on 24-hour Holter monitoring.

## Discussion

Post-PVC QTc interval prolongation has been documented in several studies. There is evidence of QTc prolongation in the first sinus beat following an extrasystole, even in structurally normal hearts. For this reason, it is recommended to measure the QT interval during stable sinus rhythm only. However, there is growing recognition that ectopic beats may reveal valuable diagnostic information in certain clinical contexts [2].

In a study by Reiffel and Reiffel, 166 individuals without abnormal repolarization findings on resting 12-lead ECG were assessed, about half of whom had no known cardiologic disease. Based on 24-hour Holter monitoring, QT prolongation following an ectopic beat was commonly observed in these subjects. However, in none of the cases did the uncorrected QT interval exceed 480 ms. In contrast, one of their patients, who presented with a clinical profile similar to ours, developed TdP after being exposed to a triggering antiarrhythmic drug. A retrospective review of that patient’s pre-drug Holter revealed a longest sinus rhythm QT interval of 450 ms, but following ectopic beats, it prolonged to 510-590 ms [5]. This pattern closely resembles that seen in our patient, whose ECG demonstrated a baseline QTc interval that significantly lengthened following PVCs.

Importantly, that study used uncorrected QT values, while in our case, we applied Bazett’s formula to adjust the QT interval for heart rate. Furthermore, while that patient carried a known LQTS type I gene mutation, the genetic variants in our patient were classified as being of uncertain pathogenic significance (VUS). According to the American College of Medical Genetics and Genomics (ACMG), a standardized framework for assessing disease causation by genetic variants includes five categories: Class V, pathogenic; Class IV, likely pathogenic; Class III, variant of uncertain significance; Class II, likely benign; Class I, benign.

While Class IV and V mutations support a diagnosis, Class III variants have unknown effects on health. A periodic reassessment of all Class III and IV variants is advised. Notably, around 20% of LQTS cases remain genetically undefined despite comprehensive testing. Furthermore, 10%-40% of genetically positive individuals may show normal or borderline QT intervals and be asymptomatic [6]. Due to this heterogeneity, clinical judgment should integrate symptoms, family history, and ECG findings when assessing patients with possible LQTS or other arrhythmia syndromes.

Significant QT prolongation in sinus cycles following a PVC reflects a temporary increase in myocardial vulnerability to arrhythmias. If the first PVC causes a marked QT prolongation in the next sinus beat, the second PVC may fall within the vulnerable period of ventricular repolarization, precipitating VT. Savelieva et al. reported that significant QT turbulence often occurs in the first sinus beat after PVCs in patients with ventricular tachycardia. During the study, they examined ECG recordings before and after stimulated ectopic beats in 40 patients with VT. The QT interval turbulence is quantified by determining the QT turbulence onset (QT TO), calculated as a relative percentage difference between the single QT interval of the first sinus beat following an ectopic beat and the mean of two QT intervals of two sinus beats preceding the ectopic beat [7]. Although we didn’t use this exact parameter to assess QT interval behavior in our patient, their observation conveyed the same idea and was consistent with our patient’s ECG finding of immediate post-PVC QTc interval prolongation.

Similarly, Haissaguerre et al. reported QT prolongation in the first QRST complex following a post-extrasystolic pause in 13 patients. These patients also showed inadequate QT adaptation during autonomic stimulation or following hydroquinidine intake. One patient had a triplet pattern typical of TdP, suggesting that poor QT adaptation after PVCs may serve as an early indicator of arrhythmogenic risk [8].

Di Biase et al. described two cases of ventricular fibrillation triggered by a sequence of events that included a pause after an extrasystolic beat, QT prolongation in the subsequent beat, and a second ectopic beat falling within the vulnerable period, despite not having a particularly short coupling interval [9]. Numerous other cases further reinforce the association between post-PVC QT prolongation and life-threatening arrhythmias, though listing all of them exceeds the scope of this review [10-11].

## Conclusions

Diagnosing cardiac electrical abnormalities such as LQTS presents significant challenges due to both clinical and genetic heterogeneity. As a result, it requires a comprehensive, multifactorial evaluation that includes symptomatology, family history, ECG analysis, and, when available, genetic testing. This case, alongside similar reports, highlights the potential importance of evaluating post-premature ventricular contraction QT interval behavior as part of that assessment. In particular, post-PVC QT prolongation may serve as an early marker of arrhythmic vulnerability, especially in patients with borderline QTc intervals or inconclusive diagnostic findings.

While this phenomenon is not yet widely recognized or routinely measured in clinical practice, its association with malignant arrhythmias suggests that it warrants further investigation. Broader awareness of post-PVC QT prolongation, coupled with future prospective studies, could enhance risk stratification in patients at risk of sudden cardiac death and inform more tailored therapeutic interventions.
